# Outcomes of Laparotomic Myomectomy during Pregnancy for Symptomatic Uterine Fibroids: A Prospective Cohort Study

**DOI:** 10.3390/jcm12196406

**Published:** 2023-10-08

**Authors:** Evgeniya Leonidovna Babunashvili, Danil Yurievich Son, Svetlana Nikolaevna Buyanova, Natalya Alekseevna Schukina, Alexander Anatolyevich Popov, Marina Alexandrovna Chechneva, Timur Alekseevich Glebov, Antonio D’Amato, Joe Haydamous, Vito Chiantera, Antonio Simone Laganà, Andrea Etrusco

**Affiliations:** 1 Gynecological Department of the Moscow Regional Research Institute of Obstetrics and Gynecology, State-Funded Health Care Facility of Moscow Region (GBUZ MO MONIIAG), 22A Pokrovka, 101000 Moscow, Russia; babounashvili@mail.ru (E.L.B.); sdanil32@gmail.com (D.Y.S.); buyanova-sn@mail.ru (S.N.B.); fluimucil@yandex.ru (N.A.S.); gyn_endoscopy@mail.ru (A.A.P.); marina-chechneva@yandex.ru (M.A.C.); glebovt@mail.ru (T.A.G.); 2 Unit of Obstetrics and Gynecology, Department of Biomedical and Human Oncological Science, University of Bari, 70100 Bari, Italy; antoniodamato19@libero.it; 3 Department of Obstetrics and Gynecology, University of Balamand, Beirut 1100, Lebanon; joehaydamous@outlook.com; 4 Department of Health Promotion, Mother and Child Care, Internal Medicine and Medical Specialties (PROMISE), University of Palermo, 90133 Palermo, Italy; vito.chiantera@unipa.it (V.C.); etruscoandrea@gmail.com (A.E.); 5 Unit of Gynecologic Oncology, National Cancer Institute—IRCCS—Fondazione “G. Pascale”, 80131 Naples, Italy; 6 Unit of Obstetrics and Gynecology, “Paolo Giaccone” Hospital, 90127 Palermo, Italy

**Keywords:** uterine fibroids, myomas, myomectomy, pregnancy, maternal–fetal outcomes

## Abstract

*Background*: The incidence of pregnant women with uterine fibroids is increasing. As they are reactive to hormonal stimuli, in some cases, uterine fibroids tend to grow during pregnancy and potentially generate symptoms with different levels of severity, causing maternal–fetal complications. In very select cases, when other treatment strategies fail to manage symptoms and there is a substantial risk of adverse pregnancy outcomes, a surgical approach during pregnancy may be considered. *Methods*: From 2016 to 2021, the data from 28 pregnant women with symptomatic uterine fibroids who underwent laparotomic myomectomy during pregnancy were prospectively collected, and operative and maternal–fetal outcomes were analyzed (ClinicalTrial ID: NCT06009562). *Results*: The procedure was carried out between 14 and 16 weeks of pregnancy. Four (14.3%) patients had intraoperative complications (miscarriages) and nine (32.1%) had postoperative complications (threatened preterm birth). Overall, 24 (85.7%) women delivered at full term (mean: 38.2 gestational weeks), more than half (*n* = 13; 54.2%) by vaginal delivery, with normal fetal weights and 1 and 5 min Apgar scores. *Conclusions*: Laparotomic myomectomy during pregnancy can be considered in selected cases for uterine fibroids with severe symptoms when other treatment options have failed and there is high risk of adverse maternal–fetal outcomes.

## 1. Introduction

Uterine fibroids (UFs) represent the most common benign tumor of the female genital tract, with an incidence ranging from 5.4 to 77% depending on various factors such as ethnicity, age, and obstetric history [1,2,3]. They consist of smooth muscle cells and fibroblasts, which secrete extracellular matrix [4]. Although most of them are asymptomatic, some UFs may be associated with pelvic pain, abnormal uterine bleeding (AUB), and infertility, depending on their location, size, and number [3,5,6,7]. Although accumulating evidence has become available in recent years, there are several issues regarding the effects and management of UFs that have yet to be clarified. Indeed, large UFs can cause compression of pelvic/abdominal organs and may undergo central colliquation due to increased vascularization, especially under the hormonal stimuli of pregnancy [8]. Nevertheless, the growth rate of UFs in pregnancy is still debated [9]. On the one hand, some data analyses failed to establish a linear trend between the hormonal status of pregnancy and the behavior of fibroids [10]; on the other hand, other authors suggested that pregnancy is associated with the growth of UFs in the majority of the cases [11]. Overall, the development of pregnancy does not always cause an increase in the size of UFs [12]. This may be due, at least in part, to non-linear increases in placental hormones (estrogen and progesterone) and endocrine and paracrine factors that play an important role in the blood supply to UFs and thus influence their growth or degeneration.

In some cases, UFs may increase the risk of adverse maternal–fetal outcomes, including severe maternal pain that does not respond to pharmacological approaches, miscarriage, preterm delivery, fetal malpresentation, premature rupture of membranes, fetal growth restriction, and placental complications [13,14,15,16]. In addition, UFs may be associated with complications during labor and delivery, e.g., abnormalities of uterine contractile activity, fetal distress, uterine atony, and postpartum hemorrhages [13,14,15,17].

Although some pieces of evidence suggest a reduction in these risks when UFs are surgically treated before pregnancy [18,19], other authors support the lack of benefits to pregnancy outcomes [20,21]. Overall, the presence of severe symptoms and signs due to UFs during pregnancy represents a challenge because pharmacological approaches to treat pain during pregnancy are limited [22]. In very selected cases, when other treatment strategies fail to manage symptoms and there is a substantial risk of adverse pregnancy outcomes, a surgical approach during pregnancy may be considered. When a surgical approach is indicated during pregnancy, a laparoscopic approach may be considered [23,24], although most of the available literature reports surgical management by laparotomy [25].

Considering these elements, the purpose of our study was to evaluate operative and maternal–fetal outcomes in women who underwent laparotomic myomectomy during pregnancy for severe symptoms/signs, aiming to reduce the risk of complications, preserve the uterus, and allow the possibility of future pregnancies.

## 2. Materials and Methods

From 2016 to 2021, we prospectively collected all the cases of laparotomic myomectomy during pregnancy (ClinicalTrial ID: NCT06009562) performed at the Moscow Regional Research Institute of Obstetrics and Gynecology (Moscow, Russia).

The Institutional Review Board of the same institute approved the design, analysis, interpretation of data, drafting, and revisions. This study conforms with the Helsinki Declaration, the Committee on Publication Ethics guidelines, and the Strengthening the Reporting of Observational Studies in Epidemiology (STROBE) Statement [18], validated by the Enhancing the Quality and Transparency of Health Research (EQUATOR) Network. The data collected were anonymized, considering the observational nature of the study, and were without personal data that could lead to formal identification of the patient. The study was not publicized. Patients did not receive any remuneration to give consent to be enrolled in this study. Each patient signed informed consent to allow data collection for research purposes. Enrolled women met the following inclusion criteria: gestational age more than 12 weeks; UFs larger than 7 cm in main diameter (when multiple myomectomy was performed, this inclusion criterion was based on the largest myoma); symptomatic UFs (rapid growth, pelvic pain, compression of nearby organs, etc.); and absence of contraindication to surgery. Patients with at least one of the following criteria were considered non-eligible: patients who refused surgery or did not sign informed consent; presence of chromosomal abnormalities and/or congenital malformations of the fetus; large UFs with cervical (retroperitoneal) location; contraindication to surgery; and absence of urgent indications for laparotomic myomectomy during pregnancy.

All patients underwent transvaginal and abdominal pelvic ultrasonography for the mapping of UFs before surgery. The need for transvaginal and abdominal approaches was determined by several factors, for instance, in very large uteri, obtaining high-quality images with only the transvaginal approach may be difficult, which is the reason why the combined method was chosen in these cases. In all patients, before surgery, fetal well-being was ascertained and additional pelvic organ pathology was ruled out and the following UF characteristics were studied: size, location, direction of growth, distance from the lower pole of the myoma to the uterine cavity, and presence of areas of colliquation or other types of degeneration and/or dystrophy. In all UFs that had suspicious intralesional areas, Doppler ultrasonography was further used to study their vasculature and exclude potential leiomyosarcoma.

Indications for laparotomic myomectomy during pregnancy included at least one of the following ones: a size of UF that prevented the proper continuation of pregnancy and/or occupied the entire small and large pelvis and/or the abdominal cavity; necrosis of the UFs; UFs causing pelvic organ dysfunction (such as unilateral or bilateral ureteral compression with hydroureteronephrosis; bowel compression with obstacle to defecation); and/or severe pelvic pain that did not respond to available pharmacological approaches.

All patients were admitted to the hospital between 7 and 19 weeks of pregnancy to be examined, undergo ultrasound, and prepare for surgery. Once admitted, all patients were given magnesium sulfate and tocolytic therapy. The tocolysis protocol consisted of micronized progesterone 400–600 mg per day and MgSO_4_ 25% − 30.0 + NaCl 0.9% − 30.0 solution infused through an infusion pump with an infusion rate of 5 mL/h. In addition, hemodynamic parameters were evaluated and corrected if necessary.

The surgical procedure was performed as subsequently described: epidural anesthesia (or endotracheal anesthesia when necessary) was used; a mini-laparotomy was performed without exteriorization of the uterus; in case of cervical and cervico-isthmic UFs, a longitudinal incision of the uterus was made; in the case of intraligamentous UFs, the round ligament of the uterus and/or the ligament of the ovary and, if necessary, the uterine vessels were cut; overall, only large UFs that prevented the development of pregnancy were removed (in order to avoid the risk of severe blood loss and, consequently, the risk of miscarriage and/or hysterectomy); sutures on the uterus were made in two layers, using long-term resorbable synthetic threads (Vicryl 00-0, Ethicon, U.S.), through the entire thickness of the myometrium, with the second layer of sutures overlapped between the ligatures of the first layer of sutures (distance between sutures on the uterus was 7–10 mm to avoid tissue ischemia); and finally, accurate hemostasis was achieved, the abdominal wall was closed in a standard fashion, and fetal wellbeing was checked by ultrasound at the end of surgery.

Clinical data were first collected from electronic medical records, and then analyzed using IBM SPSS statistics version 27. All variables were first tested for normality of distribution using histograms and Q–Q graphs. All data were normally distributed. Continuous variables are summarized as means ± standard deviation (SD) and categorical variables are summarized as frequencies (*n*) and percentages (%).

## 3. Results

During the study period, the data of 28 pregnant patients who were scheduled to undergo laparotomic myomectomy were collected. The baseline characteristics of the patients are shown in Table 1.

Nineteen (67.9%) patients were pregnant for the first time, and all of them had achieved a natural pregnancy without using assisted reproductive technologies. In 22 patients (78.6%), there was rapid growth of UFs during the first and second trimesters of pregnancy; 11 (39.3%) cases also presented altered blood flow and ultrasound evidence of changes within the lesion and elevation of inflammatory indices. There was persistent pain in the abdominal cavity and pelvis in 20 patients (71.4%); symptoms due to compression by the myoma on the surrounding organs in 5 patients (17.8%), such as acute urinary retention, tenesmus, and constipation; and a retro-amniotic hematoma in 1 patient (3.6%), which made the continuation of pregnancy dangerous. In addition, the signs and symptoms of potential miscarriage were found in 12 patients (42.8%). On average, UF-related symptom onset occurred at 9.5 weeks of pregnancy. The onset of symptoms was followed by hospitalization. The procedure was performed between 14 and 16 weeks of pregnancy, with the rationale that the risk of teratogenic effects on the fetus of many drugs decreases during this period. The interval between the onset of symptoms and surgery includes the period when diagnosis, preoperative work-up, and adequate fetal and maternal monitoring were performed.

The intraoperative anesthesia method chosen was regional block (epidural). This method provided effective and long-term anesthesia during surgery and in the postoperative period. As anesthetic, a ropivacaine solution was used, which is considered safe in pregnancy. If necessary, endotracheal anesthesia was performed. Surgical treatment in all patients involved mid-lower laparotomy. UFs at any site were removed by intracapsular myomectomy. As shown in Table 2, one UF was removed in seventeen (60.7%) patients, two UFs in eight (28.6%) patients, three UFs in two patients (7.1%), and four UFs in one patient. The mean specimen weight was 482.49 g (range 111 g–1000 g), and the mean specimen size was 10.6 cm (range 3 cm–25 cm). Although the smallest excised UF measured 3 cm, it should be noted that this was present in a patient undergoing multiple myomectomy (in this case, three UFs had been excised, the largest of which measured 10 cm). Fourteen (32.5%) of the removed UFs were at the level of the anterior wall of the uterus, seven (16.3%) of the posterior wall, nine (20.9%) of the uterine fundus, six (13.9%) of the isthmic region, and finally seven (16.3%) were intraligamentary UFs. Regarding FIGO classification, most of the removed UFs were FIGO type 6 (*n* = 8; 28.6%) and FIGO type 7 (*n* = 7; 25.0%). There were also five cases (17.8%) of FIGO type 5 UFs, five cases (17.8%) of FIGO type 4 UFs, and three cases (10.7%) of FIGO type 3 UFs. The mean operative time was 104.9 min. The average estimated blood loss was 193.6 mL, and no patients required blood transfusions.

Overall, no hysterectomy was performed; four patients (14.3%) had a miscarriage, which was the only type of intraoperative complication reported in this series. All patients undergoing intraoperative miscarriage had UFs particularly close to the uterine cavity, namely FIGO type 3 (*n* = 3, 75.0%) and FIGO type 4 (*n* = 1, 25.0%). Nine patients (32.1%) had postoperative complications: all presented a threat of miscarriage. Additionally, for three patients (33.3%), cervico-isthmic incompetence occurred, two of these patients had an obstetric pessary applied at 19 weeks of pregnancy, and one underwent cerclage at 21 weeks of pregnancy. Three patients (33.3%) also presented placental insufficiency with inadequate fetal flows found on Doppler examination. Fetal malposition was recorded in two patients (22.2%) and finally one patient underwent preterm delivery (32 weeks of pregnancy).

No intra-abdominal bleeding occurred in the postoperative period. After surgery, infusion therapy was given for 2–3 days, including protein, crystalloid solutions, and drugs that improve microcirculation and tissue regeneration. Depending on the severity of clinical signs of threatened abortion, therapy aimed at preserving pregnancy (tocolytics, antispastics, and magnesium sulfate—as described in the methods) was carried out from the first hours after surgery.

In women who did not have intraoperative miscarriage (*n* = 24; 85.7%), as shown in Table 3, more than half (*n* = 13; 54.2%) had vaginal delivery and 11 (45.8%) underwent a cesarean section. Vaginal delivery was attempted only if the UF was not located on the posterior wall of the uterus and if the thickness of the myomectomy scar, assessed by ultrasound, was more than 2 mm. None of the patients recruited in the study had uterine rupture at the time of delivery. The mean gestational age at delivery was 38.2 weeks, the mean birth weight was 3237.9 g, and the Apgar scores at 1 and 5 min were also normal in all cases.

## 4. Discussion

The current tendency to delay the age of pregnancy and the increased number of mothers over the age of 30 years have resulted in a significant increase in the frequency of pregnant women with UFs in recent years [26]. UFs can undergo significant volumetric changes during gestation, thus complicating the clinical management of these patients, and it is currently not possible for clinicians to correctly predict both the growth in the UF during pregnancy and whether it will persist, regress, or even increase in volume once delivery occurs [10]. UFs in pregnancy may therefore represent a common clinical condition which require adequate preventive strategies due to their increased risk of adverse maternal–fetal outcomes, as well as a precise therapeutic approach in the case of symptoms. Although most of these pregnancies proceed without complications, a variety of obstetric complications can be observed in approximately 30% of pregnancies with UFs [27]. The symptomatology of symptomatic UFs in pregnancy is reported in most cases as mild and often responds well to medical therapy; some very select cases, however, may require a surgical approach during pregnancy when there is substantial risk of adverse pregnancy outcomes and other therapeutic strategies fail. The most common indications for laparotomic myomectomy reported in the literature [23,28,29] correspond to those considered for the present study: acute pelvic pain unresponsive to medical therapy of >72 h, rapid growth or changes in the lesion that could conceal a neoplasm, mass compressing the pelvic organs, and a high risk of fetal adverse events (fetal compression syndrome, oligoamnios, intrauterine growth restriction, hemorrhages, and placental site abnormalities).

Overall, myomectomy in pregnancy, because of the increased uterine blood flow and volume during gestation, which increases the risk of bleeding complications and the likelihood of hysterectomy, is an operation that can predispose patients to adverse pregnancy outcomes such as miscarriage, maternal and fetal infections, preterm delivery, and uterine rupture. However, in some cases, surgical removal of the myoma is the only choice to resolve serious clinical situations [30]. In addition, patients with untreated symptomatic UFs during pregnancy seem to have a worse pregnancy outcome than patients treated surgically [31].

Recently, laparoscopy has been proposed as the first choice for abdominal and pelvic surgery during pregnancy at any gestational age because it offers better intra-abdominal magnification provided by optics, a minimally invasive approach, and earlier mobilization after surgery (critical for preventing thromboembolism) [23]. However, in cases of urgent surgery and/or large UFs, laparotomic myomectomy can be considered and it has been widely performed so far, even if the evidence regarding the feasibility and safety in pregnancy is based on a small case series [25]. In this scenario, our study, based on a relatively large cohort compared with the previous studies, confirms these elements; indeed, we found that most of the women who underwent laparotomic myomectomy delivered at full term (85.7%), with normal fetal weights and 1 and 5 min Apgar scores. Despite these findings, laparotomic myomectomy should be considered in pregnancy only when there are appropriate indications (high risk of adverse maternal–fetal outcomes due to large/symptomatic UFs), when medical approaches have failed to resolve symptoms, and by considering the risk of intraoperative miscarriage. According to most authors [9,32,33,34], the risk of developing complications related to UFs during pregnancy is directly proportional to the size of the lesions, while the exact location of the tumors in the thickness of the myometrium (submucosal, intramural, or subserosal) would seem to be responsible for different specific types of adverse events. In addition, the presence of a relatively high number of intraoperative complications was recorded in our cohort (*n* = 4; 14.3%). Although this highlights that laparotomic myomectomy in pregnant patients is not a risk-free procedure, it is important to discuss which patients experienced intraoperative miscarriages. Two (50%) of the four patients had one FIGO type 3 UF, weighing 380 g and 748 g and measuring 9 cm and 15 cm, respectively, one patient (25%) presented three FIGO type 3 UFs, the largest of which weighed 1000 g and measured 10 cm, and finally one patient (25%) had four FIGO type 4 UFs, the largest of which weighed 900 g and measured 25 cm, presenting an interior of necrotic/colliquative appearance. These types of UF can localize very close to the uterine cavity, even touching the endometrium in the specific case of an FIGO type 3 UF. This implies the need to go very deep into the thickness of the myometrium during surgery, with a very high risk of damage to placental/fetal structures and subsequent intraoperative miscarriage. It is therefore worth considering for all women with a desire for offspring the need to treat FIGO type 3 UFs, or intramural myomas particularly close to the uterine cavity. Indeed, for FIGO type 3 UFs, there is evidence to show that this type of lesion not only reduces the chances of achieving pregnancy but could be particularly dangerous once the woman is pregnant [6]. In addition, the possibility of removing this type of UF hysteroscopically would allow the patient to undergo minimally invasive surgery, with a favorable risk/benefit ratio, and to be able to attempt to achieve pregnancy within a short time after surgery [35]. Interestingly, more than half (54.2%) of the women included in our series had vaginal delivery, further stressing that vaginal delivery is a feasible option after myomectomy (especially when it is performed before pregnancy) [36]. Specifically, in an effort to allow vaginal delivery even after myomectomy is performed during the same pregnancy, an ultrasound evaluation of the myomectomy scar was performed, choosing a thickness of 2 mm as a safety margin. Overall, the operative time and estimated blood loss in our series were relatively low. Regarding the length of hospital stay, this parameter was mainly influenced by the need to perform tocolysis in certain cases (*n* = 9) with postoperative-threatened abortion. Nevertheless, no infection or deep vein thrombosis occurred, suggesting that the procedure may not be considered at high infective or thromboembolic risk.

Several limitations should be taken into a proper data interpretation: first of all, the number of enrolled women is relatively low to draw firm conclusions about the topic; secondly, we did not compare the outcomes of laparotomic myomectomy in women with severe symptomatic UFs who underwent other medical treatments or different surgical approaches or did not undergo any treatment (follow-up only). In addition, long-term follow-up after laparotomic myomectomy in pregnancy is needed to clarify future fertility outcomes and recurrence rates.

## 5. Conclusions

In the case of UFs causing severe symptoms that do not respond to other treatment strategies and result in a high risk of adverse maternal–fetal outcomes, laparotomic myomectomy during pregnancy can be considered as an option to preserve the uterus and reduce the risk of complications. Although this approach (as any other surgical procedure performed during pregnancy) is not free from risk, our data analysis highlights that more than 85.7% of the women who underwent laparotomic myomectomy during pregnancy delivered at full term, more than half by vaginal delivery. Nevertheless, considering the limitations of this study, we solicit further investigations in larger cohorts with a longer follow-up, aiming to compare this treatment with other alternatives.

## Figures and Tables

**Table 1:** Baseline characteristics of the pregnant patients planned to undergo laparotomic myomectomy.

**Baseline Characteristics**	
Age, years	31.4 ± 4.8
Body Mass Index, kg/m^2^	23.1 ± 3.3
Nulliparity, *n* (%)	19 (67.9)
**Pregnancy data**	
Natural pregnancy, *n* (%)	28 (100)
Gestational age at symptom onset, weeks	9.5 ± 2.4
Gestational age at surgery, weeks	14.9 ± 2.7
**Symptoms and signs, *n* (%)**	
Rapid growth	22 (78.6)
Pelvic pain	20 (71.4)
Compression of adjacent organs	5 (17.8)
Uterine fibroid necrosis	11 (39.3)
Retro-amniotic hematoma	1 (3.6)
Threat of miscarriage	12 (42.8)

**Table 2:** Operative data in pregnant patients who underwent laparotomic myomectomy.

Operative Data	
Number of uterine fibroids removed, *n* (%)	
1	17 (60.7)
2	8 (28.6)
3	2 (7.1)
4	1 (3.6)
≥5	0 (0.0)
FIGO classification, *n* (%)	
0	0 (0.0)
1	0 (0.0)
2	0 (0.0)
3	3 (10.7)
4	5 (17.8)
5	5 (17.8)
6	8 (28.6)
7	7 (25)
Position of the uterine fibroids removed, *n* (%)	
Anterior wall	14 (32.5)
Posterior wall	7 (16.3)
Fundus	9 (20.9)
Isthmus	6 (13.9)
Intraligamentary	7 (16.3)
Specimen weight, g (range)	482.49 (111–1000)
Specimen size, cm (range)	10.6 (3–25)
Operative time, min	104.9 ± 11.9
Estimated blood loss, mL	193.6
Transfusions, *n* (%)	0 (0)
Length of hospital stay, days	6.8 ± 0.7
Intraoperative complications, *n* (%)—miscarriage	4 (14.3)
Postoperative complications, *n* (%)	
Cervico-isthmic insufficiency	3 (33.3)
Placental insufficiency	3 (33.3)
Fetal malpresentation	2 (22.2)
Preterm labor	1 (11.1)
Threatened abortion	9 (100)
Total	9 (32.1)

**Table 3:** Maternal–fetal outcomes in pregnant patients who underwent laparotomic myomectomy.

Maternal–Fetal Outcomes	
Vaginal delivery, *n* (%)	13 (54.2)
Caesarean section, *n* (%)	11 (45.8)
Gestational age at delivery, weeks	38.2 ± 1.7
Neonatal birth weight, g	3237.9 ± 93.2

## Data Availability

An anonymized full dataset is available from the first author (E.L.B.) upon reasonable request.

## References

[1] Parker W.H. (2007). Etiology, Symptomatology, and Diagnosis of Uterine Myomas. Fertil. Steril..

[2] Stewart E.A., Laughlin-Tommaso S.K., Catherino W.H., Lalitkumar S., Gupta D., Vollenhoven B. (2016). Uterine Fibroids. Nat. Rev. Dis. Primers.

[3] Stewart E.A. (2001). Uterine Fibroids. Lancet.

[4] Laganà A.S., Vergara D., Favilli A., La Rosa V.L., Tinelli A., Gerli S., Noventa M., Vitagliano A., Triolo O., Rapisarda A.M.C. (2017). Epigenetic and Genetic Landscape of Uterine Leiomyomas: A Current View over a Common Gynecological Disease. Arch. Gynecol. Obstet..

[5] Donnez J., Dolmans M.-M. (2016). Uterine Fibroid Management: From the Present to the Future. Hum. Reprod. Update.

[6] Favilli A., Etrusco A., Chiantera V., Laganà A.S., Cicinelli E., Gerli S., Vitagliano A. (2023). Impact of FIGO Type 3 Uterine Fibroids on in Vitro Fertilization Outcomes: A Systematic Review and Meta-Analysis. Int. J. Gynaecol. Obstet..

[7] Etrusco A., Laganà A.S., di Donna M.C., Chiantera V. (2023). Uterine Leiomyomas and Problems of Deep Vein Thrombosis of the Lower Limbs: A Gynecological Point of View. Acta Phlebol..

[8] Coutinho L.M., Assis W.A., Spagnuolo-Souza A., Reis F.M. (2022). Uterine Fibroids and Pregnancy: How Do They Affect Each Other?. Reprod. Sci..

[9] Vitale S.G., Padula F., Gulino F.A. (2015). Management of Uterine Fibroids in Pregnancy: Recent Trends. Curr. Opin. Obstet. Gynecol..

[10] Vitagliano A., Noventa M., Di Spiezio Sardo A., Saccone G., Gizzo S., Borgato S., Vitale S.G., Laganà A.S., Nardelli G.B., Litta P.S. (2018). Uterine Fibroid Size Modifications during Pregnancy and Puerperium: Evidence from the First Systematic Review of Literature. Arch. Gynecol. Obstet..

[11] Mitro S.D., Peddada S., Chen Z., Buck Louis G.M., Gleason J.L., Zhang C., Grantz K.L. (2022). Natural History of Fibroids in Pregnancy: National Institute of Child Health and Human Development Fetal Growth Studies—Singletons Cohort. Fertil. Steril..

[12] Selter J.H., Price T.M., Harris B.S. (2022). Fibroids in Pregnancy: A Growing or Shrinking Issue?. Fertil. Steril..

[13] Ezzedine D., Er N. (2016). Are Women with Uterine Fibroids at Increased Risk for Adverse Pregnancy Outcome?. Clin. Obstet. Gynecol..

[14] Jenabi E., Fereidooni B. (2019). The Uterine Leiomyoma and Placenta Previa: A Meta-Analysis. J. Matern. Fetal Neonatal Med..

[15] Jenabi E., Ebrahimzadeh Zagami S. (2017). The Association between Uterine Leiomyoma and Placenta Abruption: A Meta-Analysis. J. Matern. Fetal Neonatal Med..

[16] Parazzini F., Tozzi L., Bianchi S. (2016). Pregnancy Outcome and Uterine Fibroids. Best Pract. Res. Clin. Obstet. Gynaecol..

[17] Jenabi E., Khazaei S. (2018). The Effect of Uterine Leiomyoma on the Risk of Malpresentation and Cesarean: A Meta-Analysis. J. Matern. Fetal Neonatal Med..

[18] Lu B., Wang Q., Yan L., Yu K., Cai Y. (2022). Analysis of Pregnancy Outcomes after Laparoscopic Myomectomy: A Retrospective Cohort Study. Comput. Math. Methods Med..

[19] Fagherazzi S., Borgato S., Bertin M., Vitagliano A., Tommasi L., Conte L. (2014). Pregnancy Outcome after Laparoscopic Myomectomy. Clin. Exp. Obstet. Gynecol..

[20] Loverro G., Damiani G.R., Malvasi A., Loverro M., Schonauer L.M., Muzzupapa G., Dinaro E. (2021). Myomectomy during Pregnancy: An Obstetric Overview. Minerva Obstet. Gynecol..

[21] Milazzo G.N., Catalano A., Badia V., Mallozzi M., Caserta D. (2017). Myoma and Myomectomy: Poor Evidence Concern in Pregnancy. J. Obstet. Gynaecol. Res..

[22] Vitale S.G., Tropea A., Rossetti D., Carnelli M., Cianci A. (2013). Management of Uterine Leiomyomas in Pregnancy: Review of Literature. Updates Surg..

[23] Saccardi C., Visentin S., Noventa M., Cosmi E., Litta P., Gizzo S. (2015). Uncertainties about Laparoscopic Myomectomy during Pregnancy: A Lack of Evidence or an Inherited Misconception? A Critical Literature Review Starting from a Peculiar Case. Minim. Invasive Ther. Allied Technol..

[24] Macciò A., Madeddu C., Kotsonis P., Caffiero A., Desogus A., Pietrangeli M., Paoletti A.M. (2012). Three Cases of Laparoscopic Myomectomy Performed during Pregnancy for Pedunculated Uterine Myomas. Arch. Gynecol. Obstet..

[25] Spyropoulou K., Kosmas I., Tsakiridis I., Mamopoulos A., Kalogiannidis I., Athanasiadis A., Daponte A., Dagklis T. (2020). Myomectomy during Pregnancy: A Systematic Review. Eur. J. Obstet. Gynecol. Reprod. Biol..

[26] Ciavattini A., Delli Carpini G., Clemente N., Moriconi L., Gentili C., Di Giuseppe J. (2016). Growth Trend of Small Uterine Fibroids and Human Chorionic Gonadotropin Serum Levels in Early Pregnancy: An Observational Study. Fertil. Steril..

[27] Stewart E.A., Cookson C.L., Gandolfo R.A., Schulze-Rath R. (2017). Epidemiology of Uterine Fibroids: A Systematic Review. BJOG.

[28] Joó J.G., Inovay J., Silhavy M., Papp Z. (2001). Successful Enucleation of a Necrotizing Fibroid Causing Oligohydramnios and Fetal Postural Deformity in the 25th Week of Gestation. A Case Report. J. Reprod. Med..

[29] Lolis D.E., Kalantaridou S.N., Makrydimas G., Sotiriadis A., Navrozoglou I., Zikopoulos K., Paraskevaidis E.A. (2003). Successful Myomectomy during Pregnancy. Hum. Reprod..

[30] Hasbargen U., Strauss A., Summerer-Moustaki M., Baretton G., Roth U., Kimmig R., Hepp H. (2002). Myomectomy as a Pregnancy-Preserving Option in the Carefully Selected Patient. Fetal Diagn. Ther..

[31] Algara A.C., Rodríguez A.G., Vázquez A.C., Valladares F.E.C., Ramírez P.G., Padilla E.L., Bandala C., Hernández S.L. (2015). Laparoscopic Approach for Fibroid Removal at 18 Weeks of Pregnancy. Surg. Technol. Int..

[32] Michels K.A., Velez Edwards D.R., Baird D.D., Savitz D.A., Hartmann K.E. (2014). Uterine Leiomyomata and Cesarean Birth Risk: A Prospective Cohort with Standardized Imaging. Ann. Epidemiol..

[33] Vergani P., Locatelli A., Ghidini A., Andreani M., Sala F., Pezzullo J.C. (2007). Large Uterine Leiomyomata and Risk of Cesarean Delivery. Obstet. Gynecol..

[34] Cavaliere A.F., Vidiri A., Gueli Alletti S., Fagotti A., La Milia M.C., Perossini S., Restaino S., Vizzielli G., Lanzone A., Scambia G. (2021). Surgical Treatment of “Large Uterine Masses” in Pregnancy: A Single-Center Experience. Int. J. Environ. Res. Public Health.

[35] Etrusco A., Laganà A.S., Chiantera V., Vitagliano A., Cicinelli E., Mikuš M., Šprem Goldštajn M., Ferrari F., Uccella S., Garzon S. (2023). Feasibility and Surgical Outcomes of Hysteroscopic Myomectomy of FIGO Type 3 Myoma: A Systematic Review. J. Clin. Med..

[36] Gambacorti-Passerini Z.M., Penati C., Carli A., Accordino F., Ferrari L., Berghella V., Locatelli A. (2018). Vaginal Birth after Prior Myomectomy. Eur. J. Obstet. Gynecol. Reprod. Biol..

